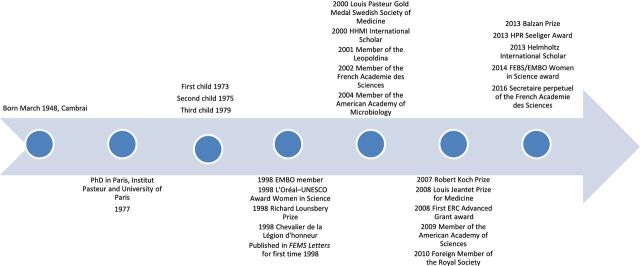# Spotlight on…Pascale Cossart

**DOI:** 10.1093/femsle/fnw215

**Published:** 2016-09-12

**Authors:** Pascale Cossart

**Affiliations:** Institut Pasteur, Unité des Interactions Bactéries-Cellules, 28 rue du Dr Roux, F75724 Paris, France

**Keywords:** intracellular pathogens, microbiology careers, omics, skills, WiSTEM, work-life balance

**Biographical summary**

**Figure 1:**
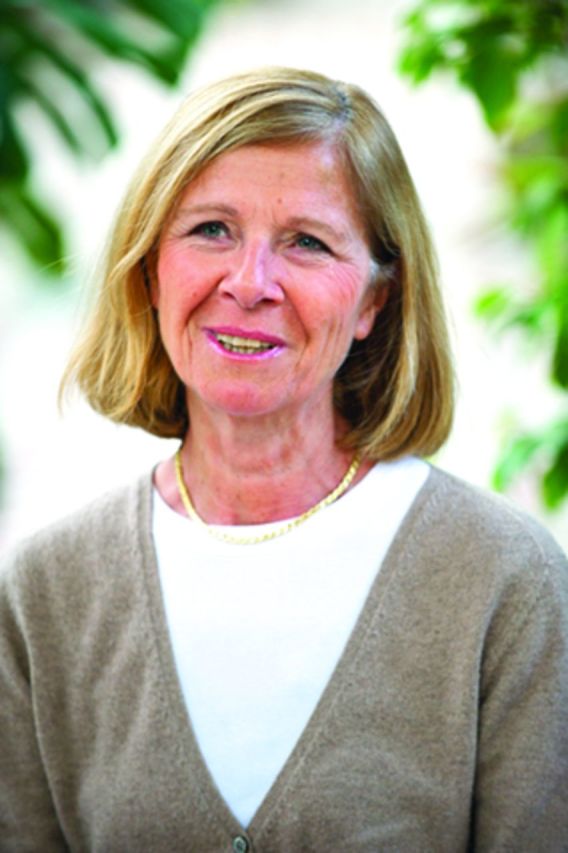

I was educated as a chemist at the University of Lille, France. After a Master of Science at Georgetown University, Washington, DC, I arrived at the Pasteur Institute in 1971, where I am still…I only interrupted this long period for 3 months in 1973 for the birth of my first daughter, 1 year to teach biochemistry in Laos in 1974 and during which my second daughter was born; my third daughter was born while I was a post doc in 1979. I am now the head of the Bacteria-Cell Interactions Unit in the Department of Cell Biology and Infection. I have just published a book entitled ‘La nouvelle Microbiology’ edited by Odile Jacob, Francois Jacob's daughter. I have also been elected Secretaire perpètuel at the Acadèmie des Sciences since January 2016.

1. **My current research** is addressing the molecular and cellular mechanisms underlying how an intracellular pathogen is behaving during life in different environments, in particular during the switch from saprophytism to virulence. We take as a model the bacterium *Listeria monocytogenes*. Our research has an impact in infection biology but also in cell biology and in fundamental biology, in particular RNA biology.
2. **I cannot say that I had decided** to have a career in microbiology. I was educated as a pure chemist in Lille. I obtained a Master degree in chemistry and started to be interested in biochemistry. I then moved to the States and at Georgetown University obtained a Master degree in chemistry/biochemistry. I then came back to France and was advised to go to the Pasteur Institute, which seemed as one of the best research places in France. At Pasteur, a succession of shifts in my interests led me from protein sequencing (remember I am originally a chemist) to DNA sequencing, to the study of DNA protein interactions. Then in 1985 – and this was quite some time after my PhD and my post doc – the director of the Pasteur Institute advised me and Brigitte Gicquel, with whom I was happily working (see below), to orient our research towards infectious diseases…It took us 6 months to decide: I thereafter focused on what appeared to me to be a potentially outstanding model, *Listeria monocytogenes*.
3. **The persons who had the most positive influence** on my career are first Jean Montreuil, who accepted me in his lab as a chemist and helped me to reorient my master towards biochemistry; then Georges Cohen, who accepted me in the Pasteur Institute and left me totally free to do what I wanted, i.e. protein sequencing; then Moshe Yaniv, who one day came back from Boston with the handout from the Maxam and Gilbert lab before publication of the paper describing how to sequence DNA – and proposed that I should come to his lab for a post doctorate and start cloning the gene corresponding to the protein I was studying and to sequence it; Brigitte Gicquel, who was interested in collaborating with me to sequence the *crp* gene – the beginning of a wonderful collaboration; John Beckwith and Richard Ebright, with whom we investigated the DNA binding site of CAP; Julian Davies, who accepted Brigitte and myself into his lab in 1986 to start investigating infectious diseases; and finally Marianne Grunberg-Manago, who has supported me much more than I was aware of…and probably played a key role in my election to the French Academy. She was the one who asked me to take over the course that she had been organising for decades in Spetses and which I am still running now.
4. **The most important skills for a microbiologist** are in my opinion the same as for any researcher: being open to any new important concept, to any new important technology and being open to the research of other people. One needs to be able to finish things that one has started but also one needs to change orientation if one realizes that one is not on the right track!
5. **My advice to early career researchers** would be ‘do what you like best, do not hesitate to switch to something else if you are no longer interested by your research theme but try to finish things by publishing your results – none of your results should stay in drawers, you will be evaluated on your papers – and more importantly, don't miss opportunities which are offered and may not come back (e.g. independence), even if in the first place they may present some inconveniencies. It is important to maintain the work–life balance. Life is short and it is important to have a nice life because work does not always bring satisfaction. Having a nice life means enjoying a family, enjoying vacations, enjoying travelling, theatre, concerts, etc. If you are a woman do not calculate too much when is the best moment to have children, it will always be difficult! Try to combine things…
6. **The greatest challenge microbiologists** are facing today is the risk of believing that omics will bring the solution to all their problems. It is absolutely not true. You need global and large scale approaches, but one needs to think, and think even more than before!

## OVERALL EXPERIENCE AS A MICROBIOLOGIST

It has been really amazing to see how progressively over the years microbiology, which had been for a while considered as an old fashioned and no longer interesting field, is now becoming again one of the most blooming disciplines in biology.

## IMPORTANT PUBLICATIONS

Kocks C, Gouin E, Tabouret M, Berche P, Ohayon H and **Cossart P**. *L. monocytogenes*-induced actin assembly requires the *actA* gene product, a surface protein. *Cell* 1992;**68**:521–31.

Mengaud J, Ohayon H, Gounon P, Mege R-M and **Cossart P**. E-cadherin is the receptor for internalin, a surface protein required for entry of *L. monocytogenes* into epithelial cells. *Cell* 1996;**84**:923–32.

Lecuit M, Vandormael-Pournin S, Lefort J, Huerre M, Gounon P, Dupuy C, Babinet C and **Cossart P**. A transgenic model for listeriosis: role of internalin in crossing the intestinal barrier. *Science* 2001;**292**:1722–5.

Glaser P, …, Wehland J and **Cossart P**. Comparative genomics of *Listeria* species. *Science* 2001;**294**:849–53.

Johansson J, Mandin P, Renzoni A, Chiaruttini C, Springer M and **Cossart P**. An RNA thermosensor controls expression of virulence genes in *Listeria monocytogenes*. *Cell* 2002;**110**:551–61.

Veiga E, Guttman JA, Bonazzi M, Boucrot E, Toledo-Arana A, Lin AE, Enninga J, Pizzaro-Cerdá J, Finlay BB, Kirchhausen T and **Cossart P**. Invasive and adherent bacterial pathogens co-opt host cell clathrin for infection. *Cell Host Microbe* 2007;**15**:340–51.

Toledo-Arana A, Dussurget O, Nikitas G, Sesto N, Guet-Revillet H, Balestrino D, Loh E, Gripenland J, Tiensuu T, Vaitkevicius K, Barthelemy M, Vergassola M, Nahori MA, Soubigou G, Régnault B, Coppée JY, Lecuit M, Johansson J and **Cossart P**. The *Listeria* transcriptional landscape from saprophytism to virulence. *Nature* 2009;**459**:950–6.

Mostowy S, Bonazzi M, Hamon MA, Tham T-N, Mallet A, Lelek M, Gouin E, Demangel C, Brosch R, Zimmer C, Sartori A, Kinoshita M, Lecuit M and **Cossart P**. Entrapment of intracytosolic bacteria by septin cage-like structures. *Cell Host Microbe* 2010;**8**:433–44.

Ribet D, Hamon M, Gouin E, Nahori MA, Impens F, Neyret-Kahn H, Gevaert K, Vandekerckhove J, Dejean A and **Cossart P**. *Listeria monocytogenes* impairs SUMOylation for efficient infection. *Nature* 2010;**464**:1192–5.

**Cossart P**. Illuminating the landscape of host-pathogen interactions with the bacterium *Listeria monocytogenes*. *Proc Natl Acad Sci U S A* 2011;**108**:19484–91.

Stavru F, Palmer AE, Wang C, Youle RJ and **Cossart P**. Atypical mitochondrial fission upon bacterial infection. *Proc Natl Acad Sci U S A* 2013;**110**:16003–8.

Mellin J-R, Koutero M, Dar D, Nahori M-A, Sorek R and **Cossart P**. Riboswiches. Sequestration of a two-component response regulator by a riboswitch-regulated noncoding RNA. *Science* 2014;**345**:940–3.

Quereda JJ, Dussurget O, Nahori MA, Ghozlane A, Volant S, Dillies MA, Regnault B, Kennedy S, Mondot S, Villoing B, **Cossart P** and **Pizarro-Cerda J**. Bacteriocin from epidemic Listeria strains alters the host intestinal microbiota to favor infection. *Proc Natl Acad Sci U S A* 2016;**113**:5706–11.

**Figure 2:**